# Community perceptions on domestic violence against pregnant women in Nepal: a qualitative study

**DOI:** 10.3402/gha.v9.31964

**Published:** 2016-11-22

**Authors:** Kunta Devi Pun, Jennifer J. Infanti, Rajendra Koju, Berit Schei, Elisabeth Darj, on behalf of the ADVANCE Study Group

**Affiliations:** 1 Kathmandu University School of Medical Sciences, Kathmandu University, Dhulikhel, Kavre, Nepal; 2 Department of Public Health and General Practice, Norwegian University of Science and Technology, Trondheim, Norway; 3 Department of Obstetrics and Gynecology, St. Olav’s Hospital, Trondheim, Norway; 4 Department of Women’s and Children’s Health, Uppsala University, Uppsala, Sweden

**Keywords:** domestic violence, pregnant women, perception, focus groups, Nepal

## Abstract

**Background:**

Globally, knowledge of health sector options to respond to domestic violence during pregnancy is increasing, but this topic is under-investigated in Nepal. This gap affects the provision of adequate antenatal care services and understanding of factors that influence women’s willingness and ability to use available services. It is critical to know more about the social norms in a community that promote and prevent women experiencing domestic violence from seeking antenatal care.

**Objective:**

To explore community perceptions of domestic violence against pregnant women.

**Methods:**

A qualitative study was conducted in Dhulikhel municipality, involving 41 men and 76 women in 12 focus group discussions in different gender and family role separated groups. The interviews were recorded, transcribed in verbatim, and analyzed using content analysis. A socio-ecological model was used as a theoretical framework to illustrate linkages between individual, relationship, community, and societal influences on perceptions of domestic violence during pregnancy.

**Results:**

The community recognized different forms of violence during pregnancy threatening women’s physical and psychological health and presenting obstacles to seeking antenatal care. Some types of culturally specific violence were considered particularly harmful, such as pressure to give birth to sons, denial of food, and forcing pregnant women to do hard physical work during pregnancy, which may leave daughters-in-law vulnerable to domestic violence in extended families. A culture where violence is normalized and endurance and family reconciliation are promoted above individual health was perceived to cause women to tolerate and accept the situation. Participants suggested actions and strategies to address continuing violence, which indicated a societal transition toward increased awareness and changing attitudes and practices.

**Conclusions:**

Domestic violence during pregnancy needs to be addressed at different levels in Nepal, where women are often dependent on others for access to health care. Social norms were perceived to be shifting toward reduced acceptance of violence against women, but restrictions on women’s life options, movement, and decision-making authority were still considered impediments to pregnant women’s health.

## Introduction

The World Health Organization’s (WHO) report (1), ‘Global and regional estimates of violence against women’, concluded that 30% of women worldwide have experienced physical and/or sexual violence by partners. According to this report, the worst affected region is Southeast Asia, with a prevalence rate of 37.7% (1). The Nepal Demographic Health Survey (NDHS) also found that one-third of women aged 15–49 years have experienced emotional, physical, or sexual violence perpetrated by their spouses (2). The WHO’s ‘World report on violence and health’ defines violence as ‘the intentional use of physical force or power, threatened or actual, against oneself, another person, or against a group or community, that either results in or has a high likelihood of resulting in injury, death, psychological harm, maldevelopment or deprivation’ (3). This definition, with its inclusion of psychological harm and deprivation, aligns well with the legal definition of ‘domestic violence’ (DV) used in Nepal: ‘any form of physical, mental, sexual, or economic harm, including acts of reprimand or emotional harm, perpetrated by one person on another with whom he or she has a family relationship’ (4). The term ‘DV’ is used in place of ‘intimate partner violence’ (IPV) (5) in Nepal, as in many studies from low-income countries (6, 7). This is based on the understanding that women in these countries often live in extended families, and potential perpetrators include other family members (8–10).

Prevalence estimates of DV in Nepal range from 30% to as high as 81%, depending on the district and the type of DV assessed (11–14). According to few qualitative studies on DV in Nepal, the risk factors most associated with women’s experiences of DV include low social status, illiteracy, economic dependency, patriarchal society, alcohol abuse by husband, insufficient or unsatisfactory dowry (according to husband’s family), polygamy, husband’s extramarital affair, unemployment, and denying sex with husband (11, 15–18).

DV that occurs during pregnancy presents significant physical and psychological health concerns for women in Nepal. Physical violence experienced during pregnancy ranges from 2% among women with higher education to 10% among women who are divorced, separated, or widowed (2). Antenatal care (ANC) services are a common point of contact between pregnant women and health systems and are, therefore, considered to present a ‘window of opportunity’ for health workers to identify and respond to DV. The WHO recommends at least four ANC visits to identify health problems associated with pregnancy. In the event of any complications, more visits or admission to hospital may be necessary (19). The Government of Nepal aims for at least 80% of all pregnant women to attend antenatal clinics (20). At present, approximately 50% of women in Nepal attend four ANC visits, 6% of women attend only one visit, and 15% of women do not attend ANC at all. Urban women (72%) are more likely to have four or more ANC visits than rural women (48%) (2).

Globally, there is increasing knowledge about effective health sector responses to DV during pregnancy, but the topic remains under-investigated in Nepal, as in many other low-income settings. This gap affects the provision of adequate ANC services, as well as understanding of the wider social and contextual factors that influence women’s willingness and ability to use ANC services. To our knowledge, no previous studies have given attention to community attitudes, health beliefs, and cultural practices which may influence the health-seeking patterns and behaviors of pregnant women living with DV. Likewise, the Ministry of Health and Population (MoHP) in Nepal has not clearly addressed gender-based violence (GBV) as an issue of health policy, although a current opportunity exists to address GBV through the national Safe Motherhood Plan and ANC (21). Integrated services for survivors of GBV are however being offered on a limited basis through some hospital-based One-Stop Crisis Management Centers (OCMCs). To provide optimal ANC services to women living with DV, it is critical to know more about the social norms in the community that promote and prevent women living with violence from seeking ANC. The aim of this study was therefore to explore community perceptions of DV against pregnant women in a Nepalese context.

## Methods

### Study design

A qualitative design was chosen to explore perceptions and experiences of DV against pregnant women, which quantitative research would not be able to capture (22). We sought community perspectives in this study through focus group discussions (FGDs). The socio-ecological model of Heise (23), which is based on the models of Belsky (24) and Bronfenbrenner (25), was used as a theoretical framework to better understand the direct and indirect influences of social groups in a community on pregnant women’s health and health-seeking behavior, at individual, relationship, community, and societal levels.

### Study setting

The study was carried out in community centers in different wards (geopolitical divisions) of Dhulikhel municipality. Dhulikhel is located in the Kavre district of Bagmati Zone, Nepal, 30 kilometers east of the capital city of Kathmandu. It is the smallest municipality of Nepal in terms of population, with 3,279 households and 14,283 people. The majority (93.3%) of the population living in Kavre district are engaged in agriculture. Most inhabitants are from the Newar ethnic group (followed by Tamang, Brahmin-hill, and Chhetri). The spoken languages of the district are Nepali, Tamang, and Newari. The literacy rate of women in this district is lower (70.3%) than that of men (88.6%) (26, 27).

### Study participants

Participants were purposively invited to take part in the study by the first author, in collaboration with four female social mobilizers working in the area. Social mobilizers are a group of trusted residents with at least school-leaving education. They are selected by the community to represent them and are trained by the municipality to visit households and convene women’s and men’s groups for the purpose of sharing public information, such as health campaigns and literacy initiatives. The social mobilizers used their existing channels for disseminating public information to invite volunteers from the community to participate in the FGDs. For example, during their weekly literacy programs with women and biweekly awareness meetings with men, the mobilizers asked for available and willing volunteers for this study. We sought broad community perspectives; thus, the selection criteria for participation were open: we asked for married participants, who have been called sons, daughters-in-law, mothers-in-law, and fathers-in-law due to their family roles. Only one individual per household was included in the groups in order to maximize openness and willingness to talk without fear of reprisal or future consequences. In total, 12 FGDs were conducted with 117 participants over the course of a 3-month period in 2015 (see Table 1 for a summary of participant and group characteristics).

**Table 1:** Participants’ characteristics

Participants	Age range	Average age	Number of participants	Number of focus group discussions
Sons	18–52	31	26	3
Daughters-in-law	18–53	26	46	4
Mothers-in-law	45–82	62	26	3
Fathers-in-law	42–81	52	10	1
Mixed men and women	30–50	39	9	1
Total participants			117	12

### Data collection

The volunteer participants were divided into gender-separated groups: sons groups and daughters-in-law groups. The sons and daughters-in-law groups were also separated from the older generation, fathers-in-law and mothers-in-law groups, in order to avoid gender dominance and subordination (28). One gender-mixed group was included. Participants in this group were educated health workers living in the same community. The rationale for including a mixed group was that open and rich discussions could be obtained from mixed groups (29, 30).

All FGDs were conducted in the private rooms, where the social mobilizers normally provide informal education and conduct community meetings. At the start of the discussions, participants were informed about the aim of the study; they were also informed that they could withdraw from the FGDs at any time if they wish without giving any reason. They were instructed to share only as much information as they felt comfortable having other participants hear them. The researchers introduced themselves as coming from Dhulikhel Hospital to study women’s health.

A semi-structured topic guide was used to conduct the FGDs. The topic guide was prepared by reviewing literature and upon extensive discussion among the authors. The topic guide was pretested for clarity of content among community members working at Dhulikhel Hospital. No major changes were made after the pretests. The topic guide covered items such as attitudes about violence within society, and specifically violence targeting pregnant women, and community responsibilities for the assistance and care of survivors. The FGDs were conducted in Nepali and audio-recorded with the permission of the participants. The first author moderated the discussions, and a research assistant observed and took notes. The moderator started the discussion by asking the participants to talk broadly about their understandings of violence against women in Nepal and then moved into the various types of violence that exist, the meaning and socio-cultural factors related to DV, and participants’ thoughts on DV specifically targeting pregnant women.

The FGDs were transcribed in verbatim from the audio-recordings and then translated into English to enable the international team of authors to participate in analysis of the material. A few transcripts were back-translated to Nepali to ensure the accuracy of the text, and no major discrepancies were found. After 12 FGDs, we felt that we had reached data saturation, as there was enough information to replicate the study (31), and no new data emerged (32).

### Data analysis

Our analytical strategy comprised the steps of qualitative content analysis as outlined by Graneheim and Lundman (33). The first author was constantly engaged in processes of reflection on her professional biography and positions as a researcher, health care provider, Nepali woman, member of a different caste/linguistic group/community, wife, daughter, daughter-in-law, and mother throughout the study. She revisited the data frequently at different points in time, keeping in mind how her multiple positions influenced the analytic processes, shaped access to information during the FGDs, and the ways community members interacted with her. The first author’s interpretations were checked with the note-taking research assistant, who was present in all FGDs, for possible misunderstandings or biases.

The main concepts in the data were identified by three authors who separately went through the transcripts line-by-line and sorted and coded the data manually into categories and subcategories. Following this initial coding, all authors read the translated transcripts and reflected on and discussed the preliminary categories and subcategories until an agreement on final content was reached. Thus, the coauthors were involved in preliminary analysis and subsequent iterative reviews of the data. The inclusion of multiple authors, with varying personal and professional backgrounds and allegiances, increased the number of ways of understanding the data and, by extension, strengthened the trustworthiness of the results and interpretations.

### Ethical considerations

The Regional Committee for Medical and Health Research Ethics of Central Norway (REK), the Nepal Health Research Council (NHRC), and Kathmandu University’s Internal Ethics Review Committee (KUIRC) approved the study. To obtain clearance from these professional bodies, the physical and psychological safety of the research participants and the researchers were thoroughly discussed, and the topic guide for the FGDs was designed to minimize potential risks.

Informed verbal consent was obtained from all participants prior to conducting the FGDs, and other standard safety protocols and guidelines were followed. For example, information about the OCMC located at Dhulikhel Hospital was provided during the FGDs as a resource for referrals for community members who came into contact with women experiencing DV. Participants were also informed that they could receive private counselling if needed following the FGDs, but no one availed of this offer.

## Results

Three key categories emerged from the FGDs on community perceptions of DV during pregnancy: threats/hazards to pregnant women’s health; obstacles to seeking care during pregnancy; and discussions about the future, particularly the impact of social and generational change on DV and community advice for addressing DV (Table 2).

**Table 2:** Categories and subcategories of community perceptions of domestic violence

Categories	Subcategories
Threats/hazards to pregnant women’s health	Heavy workload in pregnancy Denial of food in pregnancy Psychological stress due to son preference Men’s alcohol abuse Female perpetrators
Obstacles to seeking care during pregnancy	Impeded access to hospital delivery Endurance and reconciliation Normalization and futility of offering assistance
The future: impact of social and generational change on DV and community advice	Increased awareness and changing attitudes and practice Engaging family members to address DV during pregnancy Responsibilities of the community Social rehabilitation of survivors

### Threats/hazards to pregnant women’s health

The study revealed that experiences of physical abuse during pregnancy, such as hitting or kicking, were considered infrequent, but nevertheless occurred. Culturally specific forms of violence, such as forcing pregnant women to do heavy lifting and hard physical work, and denial of food, were perceived forms of combined physical and emotional abuse of pregnant women by their family members, especially perpetrated by mothers-in-law. The effects of emotional violence experienced by pregnant women, such as bullying, belittling, threats and mental stress related to dowries, gender expectations, and son preferences, were discussed and considered particularly detrimental during pregnancy. Excessive consumption of alcohol and lack of education were attributed to violence in pregnancy perpetrated mainly by husbands. Women suppressing women and women planting the ‘seed’ of a quarrel were also expressed by the participants.

#### Heavy workload in pregnancy

In Nepal, marriage before the age of 18 remains a persistent problem that affects children, especially girls. The community members in this study reported that new brides are relocated to their husband’s homes upon marriage and have little power in their new households. This makes them vulnerable to numerous forms of DV perpetrated by their husbands and in-laws. Similarly, in the discussions, a perception among community members was that daughters-in-law are treated as another ‘pair of hands’ for housework in their new families, which was considered a form of emotional abuse:

When I got married I was just 16 years, I was a child. I didn’t know anything. My husband was also only one year older than me, just 17 years. My mother didn’t want me to marry in childhood but my in-laws requested the marriage with the intention of getting someone to help cook meals for their family. But, later, they blamed and insulted me, saying I was from a beggar family. (FGD, daughters-in-law)

Participants discussed that women are pressured to bear children as soon as they are married but, paradoxically, when they become pregnant, they are often accused by their in-laws of conceiving in order to escape from the burdens of housework. As one participant mentioned, this can lead to justification for physical violence at home:

People here adhere to the concept of bringing a daughter-in-law into a home for household work. If she gets pregnant then the older people think she wants to avoid work. At that point, physical violence occurs and hampers household work. (FGD, mixed men and women)

Pregnant women were also compelled by their families to work hard outside the house, typically planting rice paddies and carrying hay in the fields. The women, especially mothers-in-law, felt that since they had worked hard when they were pregnant, their daughters-in-law should do so too. Lack of awareness and education that pregnancy places special demands on the energy requirements and physical capacity of women could contribute to this perception. Being forced to perform hard physical labor was considered a type of DV by both the women and men in the FGDs:

Looking at conditions today, it is not like in the past. No one used to care, no matter how heavy an object one carried, how hard one worked. I delivered at home, gave birth to four children, gave birth after working all through the day. Not even once went to the hospital. (FGD, mothers-in law)

#### Denial of food in pregnancy

Community members reported restrictions of food intake during pregnancy. This may be due to poverty or due to abuse by mothers-in-law, and this caused significant emotional distress, besides having physical repercussions:

Nobody may have suffered violence like me. My mother-in-law used to weigh the fodder I carried before giving me food. She used to give me one handful [showing her hands] of dhindo [thick porridge of water and flour] if she could carry the fodder I brought and two handfuls of dhindo if she couldn’t carry the fodder. (FGD, mothers-in-law)

#### Psychological stress due to son preference

The discussions revealed that violence during pregnancy was mostly psychological, as in pressure and taunting to give birth to a male child. In Nepalese society, a deep-rooted preference exists for male children over female children regardless of educational or economic status or location of residence, urban or rural. Participants felt that son preference is rooted in culture, traditions, and patriarchal norms and cannot be easily changed:

This boy and girl issue is a bizarre thing. Even educated families also wish for a son. My younger sister is a graduate level educated woman and is now a writer living in Australia. She also wished for a son. (FGD, mixed men and women)

Women mentioned preferring to have a son because they feared daughters would suffer discrimination in life:

Now we can find out whether it is a son or a daughter at the check-up so the husband can suggest aborting if it is a girl again … If the wife accepts the suggestion of the husband everything will be fine, but if she refuses to listen, then there will be tension … The tension may cause abortion sometimes. (FGD, daughters-in-law)

We see such cases sometimes in the labor room of a husband being like in a foreign country or not accompanying his wife to the hospital even, and torturing his wife on the phone then, saying that she need not return home if she delivers a baby girl. Women cry a lot due to that pain – more than the contraction pain … Moreover, if she already has one or two daughters, we cannot imagine how much she is tortured. (FGD, mixed men and women)

Participants in the focus groups also discussed that pregnant women felt powerlessness to address the injustices they experienced:

Women feel insecure because they are brought up like that. They are not allowed to climb up a tree or cross the river. They are not allowed to walk outside after 7 pm. So they have the psychology of feeling insecure. I have seen that if a woman’s family is broken, even the maternal family will not look after her. She will become homeless. So she will rather suppress [her desires] and try to remain within her husband’s house without a word. This way she will be abused mostly by her mother-in-law and sisters-in-law … Women, whether educated or not, feel they are dependent upon others. It is our culture that has taught women to be dependent on males. (FGD, mixed men and women)

If we speak they [our husbands] will oppress and beat us even more … what can we do? We are helpless. (FGD, mothers-in-law)

#### Men’s alcohol abuse

The participants perceived the consumption of alcohol by husbands as one of the main causes of DV. The discussions concluded that when men are drunk, verbal and physical disputes between husbands and wives are more likely to occur:

Drinking, coming home and shouting at the wife! This happens with those who drink … most of the time the quarrel at home is started by those who come home heavily drunk. They seek reasons to argue. They feel very powerful. (FGD, daughters-in-law)

#### Female perpetrators

Women were also considered as perpetrators of violence during pregnancy. Different opinions were discussed according to the age and gender of the participants. Older women perceived younger women as abusers:

Women themselves accuse each other of being witches, thieves and what not. Women repress women … earlier a daughter-in-law used to fear the mother-in-law but now it’s the mother-in-law who fears the daughter-in-law. No matter how much a mother-in-law loves her daughter-in-law, she never feels that the mother-in-law is like her own mother. (FGD, mothers-in-law)

Conversely, daughters-in-law accused mothers-in-law of DV during pregnancy:

In pregnancy women feel sluggish and don’t feel like working. So they are shouted at, scolded for not working. The mother-in-law scolds more than the husband and the father-in-law. (FGD, daughters-in-law)

Men agreed that mothers-in-law are the cause of violence experienced by their daughters-in-law:

Firstly women are the reason for violence against women, which men may not notice. Mothers-in-law don’t say anything to sons and keep on fighting with daughters-in-law. (FGD, sons)

Most often this type of violence on women is committed by the mother-in-law. The mother-in-law provokes the son and her husband about the dowry. (FGD, fathers-in-law)

### Obstacles to seeking care during pregnancy

The community members perceived the existence of direct and indirect causes which acted as obstacles to seeking care during pregnancy. Impeded access to hospital delivery was considered a direct cause of failure to seek care, while the culture of endurance, reconciliation, and believing DV to be a ‘normal’ phenomenon was seen as an indirect cause to care-seeking.

#### Impeded access to hospital delivery

Participants discussed pressure from in-laws to avoid going to hospitals for childbirth as a form of DV. Most women in the FGDs knew hospital births to be safer than homebirths, but the younger generation, both pregnant women and their husbands, felt they were not entitled to make autonomous decisions:

Some don’t go for [antenatal] check-ups because they don’t know about it, while others don’t go despite being aware that they should because their mothers-in-law question the value of the check-ups. The mothers-in-law say, ‘Why should you go to the hospital? The cost incurred while giving birth at the hospital could be used to manage expenditures in post-pregnancy’. (FGD, daughters-in-law)

This kind of controlling behavior from mothers-in-law was perceived as compromising pregnant women’s health, including the health of the unborn child.

#### Endurance and reconciliation

Arranged marriages by parents are common in Nepal, divorce is uncommon, and the majority of people live in extended family households. As such, one of the most common coping strategies for victims of DV is to ‘endure it’ and focus on restoring domestic harmony. Participants expressed that, after marriage, women should try to forget about their parents’ houses and instead think of their husbands’ homes as their own homes; they should do ‘what is needed’ at their husband’s houses. Domestic conflicts should remain within the household too, as these are considered private family matters, and thus can act as a factor hindering pregnant women from seeking ANC:

After marriage, a woman’s husband’s house becomes like her maternal home … While taking care of this home [husband’s house] she should treat it like her own. Women should be aware of this responsibility … Some women say, ‘I have suffered after coming here. My maternal home is this and that’. They need to understand it is their own fate, like a lottery, some landed in better houses and some in houses where they have to suffer a little. (FGD, sons)

Women in the FGDs felt that reconciliation was the only viable strategy in response to DV:

The husband and the wife must reconcile, otherwise how can the family function? … It is not okay to speak against the husband even if he is abusive. The family needs to counsel him and try to live in harmony. (FGD, daughters-in-law)

Society is unable to decrease violence. If we speak, they [the abusers] will oppress and beat us even more … But if one stays quiet, then it will be alright … The other [the victim] has to tolerate it. Only then will there be no quarrels … If the son and the daughter-in-law respects [sic] the mother-in-law, it will be good … It will worsen if one thinks s/he is better than the others. (FGD, mothers-in-law)

Women’s decisions to endure violence were also described as linked to their lack of independent resources, such as social and economic security:

When you go to the government legal office for women, they keep your case in waiting for many days. Then they … may punish the person who committed violence against us, but they cannot keep us safe for life, giving us our share of the property and a job. Instead they take us back to the community and the same house. The family will torture us even more then for reporting about them. For this reason women prefer to bear violence as far as possible. (FGD, daughters-in-law)

Those [women] who have good economical and physical strength will have more power, and those who have bad economic conditions are suppressed … One woman came to the OPD [out-patient department of a hospital] giving a history of being beaten by her husband … She preferred not reporting her husband because she feared further consequence. She had to go back and live in her husband’s house again and feared getting beaten twice as much if she made a formal report. (FGD, mixed men and women)

#### ‘Normalization’ and futility of offering assistance

The normalization process makes pregnant women see DV as a commonplace situation. This means that they may not consider seeking help for DV at ANC. This also means that neighbors may not intervene in what are considered ‘family matters’. A metaphor mentioned in the FGDs by both men and women was that fights between husbands and wives are common, inevitable, and insignificant, like a ‘fire in a haystack, domestic quarrels start fiercely and end quickly’. Another implication of this metaphor is the belief that couples will immediately patch up, with a problem only becoming complex if a third party intervenes. Overall, there was a striking tendency in the community to justify DV as a normal phenomenon, which might reflect the reality of limited alternatives for abused pregnant women:

Haystack fires burn fiercely for a short time, then die out quickly … It is normal to quarrel but, as a human being, we have to reconcile … Husbands and wives may fight the whole day but then immediately patch up. It is outsiders who are made to be the enemy. Those who speak to the couple about the situation become ‘the bad ones’. (FGD, daughters-in-law)

The men expressed their attitude that violence is relevant and should be used:

Beating is also necessary. First we should try to make her understand, if she does not understand then [the husband] should beat her. [Ha! Ha!] (FGD, sons)

It was clear from the group discussions that it is complicated for other people in the community, informal helpers, such as friends or neighbors, to assist women experiencing DV. Interventions by others were often described as counterproductive, for example, leading neighbors to become enemies or victims of insult too. A dialogue in one of the focus groups between five young women illustrated this reality:

Here people want to keep these things [domestic quarrels] secret. When outsiders approach, they pretend that nothing has happened. The husband and wife try to reconcile and hope others will not come to know about it. But once the people go away, the quarreling starts again (P1). And then they blame the community for not helping. How can we help if it is like this (P2)? Two or three days later they make up and we end up being the guilty ones (P3). Help should be given to them [abused women] but if you ask them, ‘why you are quarrelling?’ they will instead accuse us saying, ‘why are you taking interest in our family matter? Just go away quickly’. Then what can we do? After that, can we say anything? I have told one husband that, ‘It is not only the matter of your family, it is our community’s matter too, so don’t do that. It affects the community also’…He just yelled at me (P4). Yes, it is very difficult. If we go to a house and say something he will accuse us of instigating his wife (P5). (FGD, daughters-in-law)

Participants also discussed how providing assistance to women experiencing DV is challenging for older men. When older men attempt to help younger women at home or in the community, such as their daughters-in-law, they are often accused of having an affair with the young women. In turn, this allegation becomes the main cause for further quarrels at home. This type of rumor also spreads quickly throughout the community and increases men’s reluctance to intervene to help or support their daughters-in-law:

I will tell my wife to love our daughter-in-law and not to ask her to carry or lift heavy objects in pregnancy. In such situations though my own wife could think negatively and say things like, ‘My husband is attracted to her [daughter-in-law] and he doesn’t tolerate me’ … When the son returns, the mother tells tales about the father-in-law and daughter-in-law … The son is turned against his father, and the quarrels flare up in the entire family … When helping a daughter-in-law these kinds of accusations happen and they spread all over the village. (FGD, fathers-in-law)

### The future: impact of social and generational change on DV and community advice

Community members perceived that violence is less common today than it was in earlier times. This was reflected by older women talking about ‘their times’. Participants also expressed that nowadays people are more aware of the impact of DV on pregnant women due to increased knowledge about women’s rights, and resulting changes in social beliefs, practices, and attitudes.

#### Increased awareness and changing attitudes and practice

During the discussions, participants of both genders and all ages acknowledged that women today have more knowledge and awareness of health requirements during pregnancy, such as reducing the amount of hard physical labor and attending regular checkups. They felt that this could be the result of women’s increased education and subsequent improvements in self-confidence and changes to social norms and gender roles, such as women having more authority about their earnings and recognizing the need for independent financial security:

Some of the new knowledge and awareness is about women’s rights to decide about family, rights about property, rights to stay physically and mentally healthy, rights for education. There are lots of others. They may be about freedom. Many may know about these rights today but cannot implement them in their real lives. (FGD, sons)

Changing concepts about equality between men and women, women’s empowerment, women’s rights, and laws for punishing perpetrators of DV were discussed in the FGDs:

Men and women are (like) equals today. Both men and women are doing the household work almost equally now … The husbands should work and we also need to work. It is not enough that only the husband works … Empowerment of women is taking place now. There is a strong law to protect women [from domestic violence]. One shouldn’t keep silent now. (FGD, daughters-in-law)

We should encourage male partners to understand their responsibility to assist their wives with the household work themselves, rather than somebody asking him to do this work … It should come spontaneously and shouldn’t be regulated by somebody’s law and order … Yes, the changes should be in [our] thinking. (FGD, mixed men and women)

Changes in attitudes regarding violence in pregnancy emerged from the discussions. It was noted that in some Nepali cultures, women received more attention during pregnancy than in other times; also, female survivors of DV have started to retaliate now:

Some societies in Nepal have such a culture that women are highly cared for during pregnancy. Their nutrition and food is looked after. (FGD, mixed men and women)

Now-a-days, if a husband slaps a wife once, she will slap him back thrice. Only in the past were wives beaten … Earlier, the wife used to tolerate the beatings from the husband, but now the wife retaliates. (FGD, mothers-in-law)

Other changes in social practices were discussed in the focus groups and attributed to a reduction in DV, such as the education of girls and evolving cultural traditions which afforded women more rights:

In our grandfathers’ time they said that only a son could light the funeral pyre [the last rite to be performed on a dead body]. But now daughters have started lighting the funeral pyres and taking part in the funeral processions. (FGD, daughters-in-law)

To bring changes, I must not only teach these things to my daughter. I will educate her and make her independent. Only then will I marry her off. Thus she will be able to earn her living in her house. I must also teach her that if somebody mistreats her, she shouldn’t tolerate it. She can work and live on her own [if needed] … We should provide them [our daughters] with the opportunities to get skills, education from childhood, and make them able persons. (FGD, mothers-in-law)

Participants perceived that preventing DV is important, particularly by organizing awareness programs at the community level to encourage individuals to assume responsibility and increase awareness of relevant laws to protect survivors and punish perpetrators. However, the details of the legal protections from DV were not perceived as general knowledge in the community, nor were the legal means for the punishment of perpetrators. Participants felt that it could be possible to achieve sustainable changes in attitudes and behaviors by engaging community-based committees in the prevention and protection of women and children:

If we gather the community together for formal training programs, they will feel the responsibility and act accordingly … There may only be ten people in one hundred who change, but if one hundred out of one thousand change after training, the country will develop very soon. (FGD, sons)

Family law has developed in foreign countries to solve this [DV]. We too can develop legal awareness with the help of paralegal committees. (FGD, mixed men and women)

#### Engaging family members to address DV during pregnancy

Mixed arguments were expressed in the discussion groups regarding domestic responsibility for controlling violence; participants expressed that either mothers-in-law or fathers-in-law should assume this role. The perception that familial authority and control is vested in the eldest male, usually the father-in-law in the household, was prevalent. However, control over domestic affairs was considered to lie just as well with the mother of the son, who exercises decision-making authority over household tasks, resources, and the activities of the sons’ wives. Given the mother-in-law’s authority, participants felt that she should also be responsible for controlling DV in a household:

It is the mother-in-law’s responsibility to manage the matters of a family inside the house. Though food and clothing are all managed by the father-in-law, he is out during the day. He is present in the house only in the mornings and the evenings unless he is too old to go out. It is the woman [mother-in-law] then who has to look after the house properly in the day time. (FGD, fathers-in-law)

Other participants held the opposite belief:

We shouldn’t blame the mother-in-law only. The father-in-law also needs to conduct himself properly. He must look after the daughter-in-law and control his wife, the mother-in-law. He should remind his wife that she is also a daughter of someone … To stop the suffering of a daughter-in-law, the father-in-law must deal with the mother-in-law properly. (FGD, fathers-in-law)

Fathers-in-law in the FGDs recommended engaging themselves in supportive roles to assist women experiencing DV:

The whole family must be ready to help her. In this activity the father-in-law must take a more active role, like by asking his wife not to make the daughter-in-law do heavy work. We can help by carrying water pots … The mother-in-law knows that during pregnancy it is difficult for a woman as she herself has experienced pregnancy. Therefore, it is the mother-in-law who should arrange that the pregnant daughter-in-law has comfort. (FGD, fathers-in-law)

Given that son preference was perceived as one of the most significant reasons for DV during pregnancy, participants in the FGDs suggested equal treatment of girls and boys as a strategy to address DV:

We should educate both fathers and mothers about equal treatment of their children. I think mothers have the main role. The statistics have shown that if the mother is educated and her career is good, her daughters will be good too, get proper education, be financially secure, be empowered, treat her children equally. (FGD, mixed men and women)

#### Responsibilities of the community

Despite the challenges of intervening in domestic disputes, community members expressed that it was among their responsibilities to help abused women. They mentioned that pregnant women experiencing abuse could be helped to fulfill their household chores, be given proper food or taken to antenatal checkups. If needed, participants stated they could help by calling the police or for an ambulance, and they felt there was a need to inform people of the consequences of DV through awareness programs especially targeting in-laws, or on more general topics such as women’s empowerment. The study participants also expressed that abused women should speak up about their problems:

We can do a lot to help women living with DV. We can organize some interaction programs. Since it [violence] is due to lack of education, mothers-in-law and fathers-in-law can be brought together to be given awareness from people like you [nurses, researchers] who know more about it. We can provide them with training. If they could be trained, then they could recognize that they are doing wrong and they will know that their sons and daughters-in-law are in a violent relationship. (FGD, sons)

In my opinion, we, the community members, should encourage preventive measures, particularly focusing on pregnant women. Education should be given to both mothers-in-law and daughters-in-law together. Though the mother-in-law may have delivered many children, she needs education relevant to the present context. We can talk about violence and its consequences with the husband and wife together too. Many misconceptions about women being responsible for the sex of the child can be cleared up that way. Several cultural acts that result in women’s oppression … should be discouraged. (FGD, mixed men and women)

#### Social rehabilitation of survivors

In the mixed FGD with young, well-educated men and women, there was considerable discussion about how to make longer term rehabilitation possible for female survivors of DV during pregnancy. This related primarily to concerns about the security of women after they leave support centers providing care to victims of DV, such as the OCMC at the local hospital:

Women get treatment immediately in case of physical assault. But we should think about their rehabilitation too … We can make a confidential filing system [at the OCMC] so that no one will know about the cases. Some days ago one victim’s father fought with the hospital ward sisters for the patient files…we have to make a system such that perpetrators and victims will be anonymous [on paper]. (FGD, mixed men and women)

## Discussions

Our study shows that the community perceives threats to pregnant women’s health due to existing DV and consequent obstacles to seeking ANC. The community members also expressed that social and generational change has impacted DV, making it less common than it was in earlier times. Consistent with Heise’s adaptation of the ecological model, the results show linkages between individual, relationship, community, and societal influences that affect understandings of DV (23). Hence, this model provides a comprehensive framework for illustrating community perceptions of DV during pregnancy in our study setting.

### Individual level

Women are in a vulnerable position in the family as they are dependent on their husbands and mothers-in-law. The community members perceived that daughters-in-law are treated as another pair of hands for housework in their new families after marriage and they are expected to bear children soon after marriage. We agree with a previous Nepalese study, stating that those women are disproportionately affected compared to men (15).

The United Nations Children’s Fund (UNICEF) has recognized that from a very young age in Nepal, confidence and self-esteem through autonomy and self-sufficiency are not cultivated in girl children; rather, self-denial, gentleness, sacrifice, and unassertiveness are encouraged in their upbringing (34). The participants in our study expressed that culture has taught women, whether educated or not, to depend on others. Inability to make decisions about their own health and mothers-in-law impeding access to health for pregnant daughters-in-law, combined with the lack of knowledge, may lead to less awareness about birth preparedness or complication readiness, resulting in increased maternal and neonatal mortality and morbidity (35).

The perception that pregnant women feel powerlessness to address experienced injustices was expressed. Consequently, a common coping strategy for survivors of DV is endurance and reconciliation. Indeed, this is often considered the only viable strategy due to lack of independent resources and social and economic security. Similar findings have been noted in studies in India and Pakistan, where survivors of IPV emphasized the necessity to endure or ‘bear’ the violence (36, 37).

### Relationship level

Attitudes restricting pregnant women from attending ANC are primarily held by mothers-in-law within the family structure. Mothers-in-law exercise control over available household resources and can prevent daughters-in-law from obtaining ANC (38). Since male involvement in maternity care is not common in Nepal (39), mothers-in-law are given more responsibility and authority for the care of pregnant women in the belief that they understand women’s issues including health problems and childbirth (40). In our study, mothers-in-law may force pregnant women to do physically hard work, deny or restrict food, and impede access to ANC. Similar findings have been observed in studies in Nepal’s neighboring country, India, where such abuse and prevention of access to health care during pregnancy were found and attributed to lack of knowledge/awareness or financial constraints from mothers-in-law (6, 10). Abuse by mothers-in-law and husbands was also discussed in a study from Bangladesh, where mothers-in-law have considerable impact on the perpetuation of DV against daughters-in-law (7).

Contradictory to these studies though, Gupta et al. found that mothers-in-law play an integral and multifaceted role in maternal and infant health in rural northern Ghana (41). Another study from India supported the fact, suggesting that the relationship between mothers-in-law and daughters-in-law – while commonly thought of as an opposing, intergenerational relationship – can be mobilized to mitigate DV with increased awareness and knowledge of gender inequities (42). In our study, husbands’ alcohol abuse and GBV are commonly related, as in other studies (10, 18 41–45).

Interestingly, participants of both genders and all ages acknowledged that there is increased awareness about the consequences of DV during pregnancy and changing attitudes and practices. Women now retaliate and, with increased education, women now attend ANC care and take other initiatives to ensure self-care during pregnancy.

### Community level

As in other studies (7, 15, 16), our study revealed son preference as a significant cause for DV. Son preference, early marriage, and conflicts between daughters-in-law and mothers-in-law are reflections of subordination in patriarchal societies (46). The community in our study perceived it to be complicated for other people to intervene where DV was known because it was considered a private matter. The security of female survivors after they leave support centers was also considered a significant concern. However, equal treatment of sons and daughters was mentioned as a strategy to address DV. Changing attitudes by organizing awareness programs in the community, disseminating knowledge about relevant laws to protect survivors and punish perpetrators, engaging family members to address DV during pregnancy, and encouraging the community to take responsibility for assisting abused women were suggestions for reducing DV. At present, participants perceived that knowledge about legal protections from DV and means of punishment for perpetrators was limited. A recent study (47) suggests the engagement of men and boys in intervention actions to prevent violence against women is essential for reducing gender inequities. This illustrates a conceptual shift from treating men only as perpetrators to approaches that seek to transform the relationships, social norms, and systems that sustain DV (47). However, this was not discussed in this Nepali context.

### Societal level

Laws against DV exist in Nepal; however, according to the discussions, they are not known well enough nor followed (4). The society accepts fighting between married couples, likening it to ‘a fire in a haystack’ that burns fiercely but for a short time. DV is considered a normal phenomenon, likely reflecting the limited alternatives for abused pregnant women. Despite shifts in the attitudes of younger women and men, Nepali society is still patrilineal and patriarchal. Successful programs to reduce violence against pregnant women in a society must engage multiple stakeholders and adopt a variety of approaches due to the multiple factors underlying violent behavior.

### Trustworthiness

The *credibility* of this study was ensured by carefully describing the FGD method, systematically analyzing the data, frequently revisiting the data, and presenting the content with substantial quotes from the research participants. Furthermore, the collaboration of international researchers involved in this study, with experience of research on GBV in low-income countries, was important for gaining an in-depth understanding of the data. The first author, who is a fluent Nepali speaker, conducted all the FGDs within a 3-month period, allowing for consistency throughout the data collection process and thereby increasing its *dependability*. A clear and detailed description of the study context and setting, description of the participants, and data collection and analysis processes has been provided for the readers to improve *transferability*.

### Strength and limitations

The strength of this study is that it is the first, to our knowledge, to provide rich data on community perceptions of DV during pregnancy in Nepal. This is important as it will facilitate an improved understanding on how a community views DV in pregnancy in different perspectives. A possible limitation could be that we were not sure whether the participants shared actual perceptions or something they considered to be socially acceptable and desirable. However, while DV is a sensitive issue, we did not get the impression that participants felt constrained or concerned during the FGDs. Rather, they spoke openly and freely.

## Conclusions

The study gives voice to some of the experiences and perceptions of a population not widely represented in the health-related literature on DV, which contributes to a clearer picture of who is affected by DV during pregnancy in Nepal and how. It demonstrates that DV during pregnancy exists and may hinder pregnant women’s access to ANC. In addition, the causes of DV are multifactorial and must be addressed at different levels within society, specifically in extended families where daughters-in-law are vulnerable to DV. Equity between the sexes is particularly important, as is increased community awareness through education programs of the laws against DV in Nepal. A notable and ongoing transition in the awareness levels of the community is helping to identify actions and strategies to curb DV and contributing to a hopeful outlook for a future where social norms could shift toward reduced acceptance of violence against women. At the same time, restrictions on women’s life options, movement, and decision-making authority still need to be addressed to make significant improvements in pregnant women’s physical and psychological health.

## Paper context

Community views on domestic violence during pregnancy are under explored in Nepal. This study sought community perceptions on violence during pregnancy threatening women’s health and presenting obstacles to seeking antenatal care, through focus group discussions in gender and family role separated groups. Participants suggested actions and strategies at different levels to address violence, such as equal treatment of sons and daughters, organizing awareness programs about domestic violence laws, and engaging family members as women’s allies.
